# Potential Sabotage of Host Cell Physiology by Apicomplexan Parasites for Their Survival Benefits

**DOI:** 10.3389/fimmu.2017.01261

**Published:** 2017-10-13

**Authors:** Shalini Chakraborty, Sonti Roy, Hiral Uday Mistry, Shweta Murthy, Neena George, Vasundhra Bhandari, Paresh Sharma

**Affiliations:** ^1^National Institute of Animal Biotechnology (NIAB-DBT), Hyderabad, India

**Keywords:** *Plasmodium*, *Toxoplasma*, *Theileria*, *Babesia*, *Cryptosporidium*, host signaling pathways

## Abstract

*Plasmodium, Toxoplasma, Cryptosporidium, Babesia*, and *Theileria* are the major apicomplexan parasites affecting humans or animals worldwide. These pathogens represent an excellent example of host manipulators who can overturn host signaling pathways for their survival. They infect different types of host cells and take charge of the host machinery to gain nutrients and prevent itself from host attack. The mechanisms by which these pathogens modulate the host signaling pathways are well studied for *Plasmodium, Toxoplasma, Cryptosporidium*, and *Theileria*, except for limited studies on *Babesia*. *Theileria* is a unique pathogen taking into account the way it modulates host cell transformation, resulting in its clonal expansion. These parasites majorly modulate similar host signaling pathways, however, the disease outcome and effect is different among them. In this review, we discuss the approaches of these apicomplexan to manipulate the host–parasite clearance pathways during infection, invasion, survival, and egress.

## Introduction

The Apicomplexan parasites represent a major class of pathogens with a wide host range. They have emerged as one of the most successful intracellular parasites, which efficiently modulate the host for their survival benefits. In this review, we focus on the potential sabotage mechanisms adopted by the five well-studied pathogens of human and veterinary importance: *Plasmodium falciparum* (malaria), *Babesia bovis* (babesiosis), *Theileria annulata* (theileriosis), *Toxoplasma gondii* (toxoplasmosis), and *Cryptosporidium parvum* (cryptosporidiosis). These parasites are morphologically similar; however, variations exist in the context of host range, mode of infection, invasion, and replication inside the host (Table 1).

**Table 1:** A generalized comparative account among *Theileria, Plasmodium, Babesia, Toxoplasma*, and *Cryptosporidium* parasites.

	*Theileria*	*Plasmodium*	*Babesia*	*Toxoplasma*	*Cryptosporidium*
Cells infected	Lymphocytes and RBC	Hepatocytes and RBC	Lymphocytes and RBC	Intestinal cells	Enterocytes
Dense granules	Yes	Yes	Spherical bodies	Yes	Yes
Parasitophorous Vacuole (PV)	No	Yes	No	Yes	Yes
Apicoplast	Yes	Yes	Yes	Yes	No
Host	Animals	Human	Animals	Animals	Animals
		Animals	Humans	Humans	Humans
Invasion process	Zippering	Gliding	Gliding	Gliding	Gliding
Conoid structure	No	No	No	Yes	Yes
Vector	Tick	Mosquito	Tick	No	No
Major species	*Theileria annulata, T. parva, T. equi, T. orientalis*	*Plasmodium falciparum, P. vivax, P. ovale, Plasmodium berghei, P. knowlesi, P. malariae*	*B. bigemina, Babesia bovis, B. major, B. divergens, B. microti*	*Toxoplasma gondii*	*Cryptosporidium parvum, C. hominis, C. canis, C. felis, C. meleagridis, C. muris*
Pathogenic stage	Schizont	Schizont	Sporozoite	Tachyzoites	Trophozoite
Zoonotic	No	No except *P. knowlesi*	Yes	Yes	Yes

Beginning with transmission, *P. falciparum, T. annulata*, and *B. bovis* are vector borne; however, *C. parvum* and *T. gondii* do not require a vector and the host is infected by oocyst-ingestion (Table 1). *T. annulata* solely infects animals impacting their health and causing huge economic loss, whereas other parasites have broader host preference range. *P. falciparum and T. gondii* infections affect human health and cause mortality worldwide. On the other hand, *B. bovis* and *C. parvum* are comparatively less pathogenic with fewer reported cases of mortality and morbidity.

In this review, we epitomize the major blueprint of the pathways targeted by these parasites to sabotage the host defense mechanism for their survival and consequent disease progression.

## *Plasmodium*: The Malaria Parasite

*Plasmodium falciparum* is considered the most lethal among the *Plasmodium* species, as it accounts for serious illness and high mortality (1–5). Two hundred fourteen million new cases of malaria are reported worldwide with a 35% mortality rate reported for children below 5 years of age (6).

**Figure 1:**
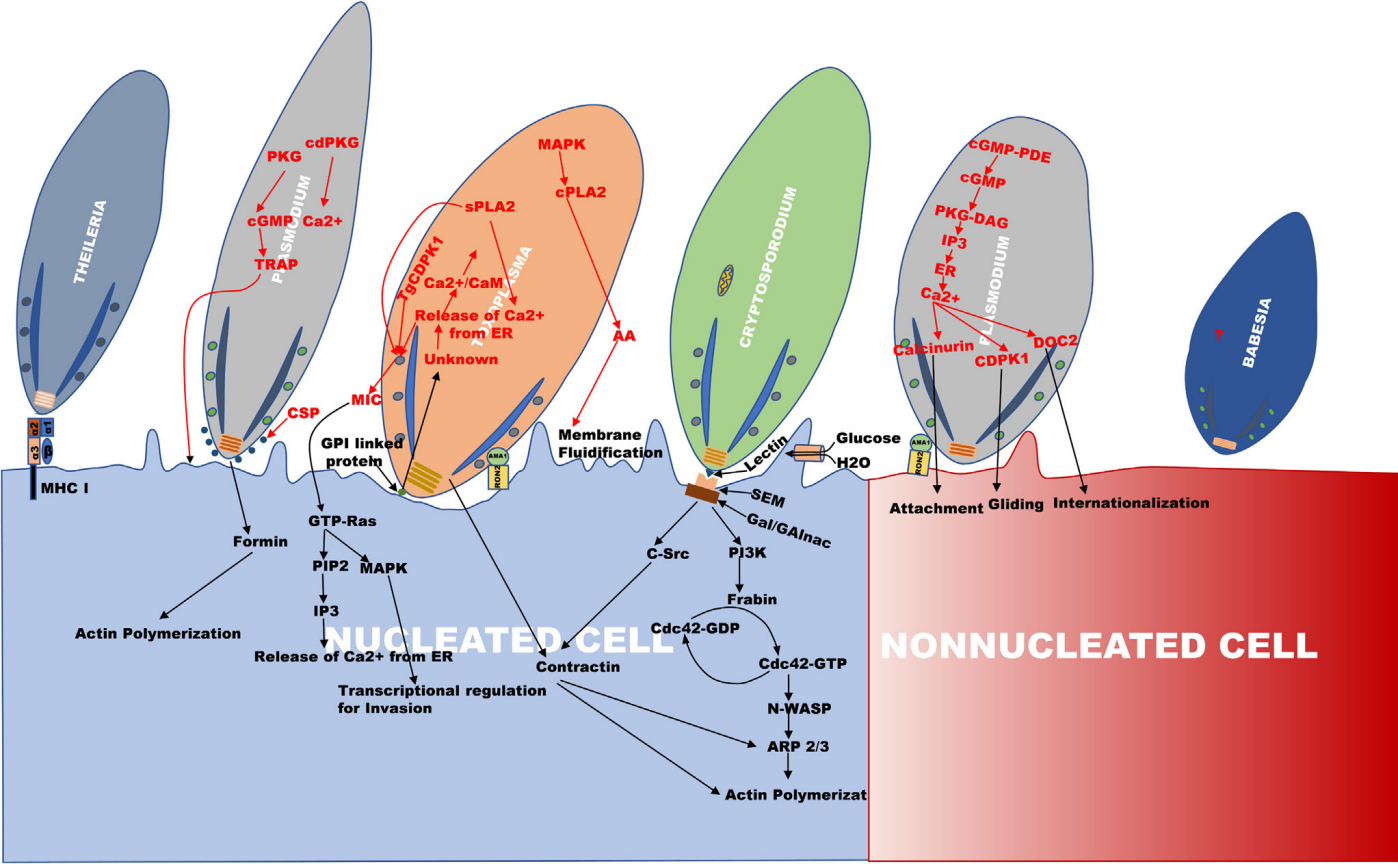
An outline of the invasion mechanism used by *Theileria, Plasmodium, Toxoplasma, Cryptosporidium*, and *Babesia*. The figure is the representation of the invasion process, which happens during internalization of *Theileria, Plasmodium, Toxoplasma, Cryptosporidium, and Babesia*. The apical region of all the parasites faces toward the cell surface for their entry. In *Plasmodium*, invasion occurs in two types of cells, erythrocyte (non-nucleated cell) and hepatocyte (nucleated cell) as compared to other parasites where invasion occurs in the nucleated cell.

Malaria transmission cycle starts with the female *Anopheles* feeding on a mammalian host. Thereafter, *Plasmodium* smartly exploits host cell machinery in numerous ways discussed hereafter to complete its life cycle (7–9). The sporozoites harbored in the salivary gland enter the host blood stream and passes on to the hepatic sinusoid (10–12). The presence of antihistamines and immunomodulators in the salivary gland secretion protects *Plasmodium* from the initial host immune response (10, 11, 13). The endothelial cell lining the liver sinusoid, guarded by kupffer cells (liver macrophages) prevents sporozoite entry into the hepatocytes (12, 14, 15). The circumsporozoite protein (CSP) of the parasite interacts with LRP-1 (low-density lipoprotein receptor-related protein) present on the kupffer cells thereby upregulating cAMP. Thereafter, cAMP mediates EPAC (exchange protein activated by cAMP) inhibition of reactive oxygen species (ROS) production ultimately suppressing the macrophage defense (15–17) (Figure 1). Simultaneously, the expression of TNFα, IL-6, and monocyte chemoattractant protein-1 (MCP-1) is downregulated and there is an increased production of anti-inflammatory IL-10 cytokine (15). The sporozoite also downregulates expression of kupffer cells MHC-1 and IL-12 to overturn their antigen presenting ability and ease infiltration of sporozoites into hepatocytes (15, 17). All these events result in the successful invasion.

Furthermore, the role of calcium (Ca^2+^) in activating various parasite proteins involved in the process of invasion, egress, motility, and cell cycle regulation has been observed (18–21). In *Plasmodium*, endoplasmic reticulum and acidocalcisomes are the major Ca^2+^ reservoirs which are also observed in *Toxoplasma*. The activation of protein kinase G (PKG) by an unknown parasitic signal during invasion or egress releases Ca^2+^ from the parasite endoplasmic reticulum mediated by cyclic guanosine monophosphate (cGMP) (20). Furthermore, phosphoinositide phospholipase C (PI-PLC) is activated by cGMP-dependent PKG which results in hydrolysis of phosphatidylinositol 4, 5-bisphosphate (PIP2) to diacylglycerol (DAG) and inositol 1,4,5-trisphosphate (IP3). The translocation of IP3 on to the ER surface causes efflux of Ca^2+^ to the cytoplasm by the formation of IP3-Ca^2+^ channel (20, 22) (Figure 1). The increase in the cytoplasmic Ca^2+^ levels activates various calcium-dependent proteases and kinases, like calcium-dependent protein kinases (PfCDPK), double C2 domain protein (PfDOC2) which induce the secretion of microneme and rhoptry proteins for cell adherence and invasion (20, 23, 24). The hepatocyte invasion of the sporozoites occurs *via* Ca^2+^-mediated activation and secretion of microneme proteins, CSP and thrombospondin-related adhesion protein (TRAP) such as Trap-like protein (TLP) (12, 15, 25) (Figure 1). The CSP secreted to the apex in association with actin covers the surface of the sporozoites and its glycosyl phosphatidyl inositol (GPI) anchored C terminus helps in the invasion of sporozoites (12, 26, 27). PfTRAP (TLP) protein interaction with actomyosin motor complex helps in gliding movement of the parasite (15, 25, 28).

**Figure 2:**
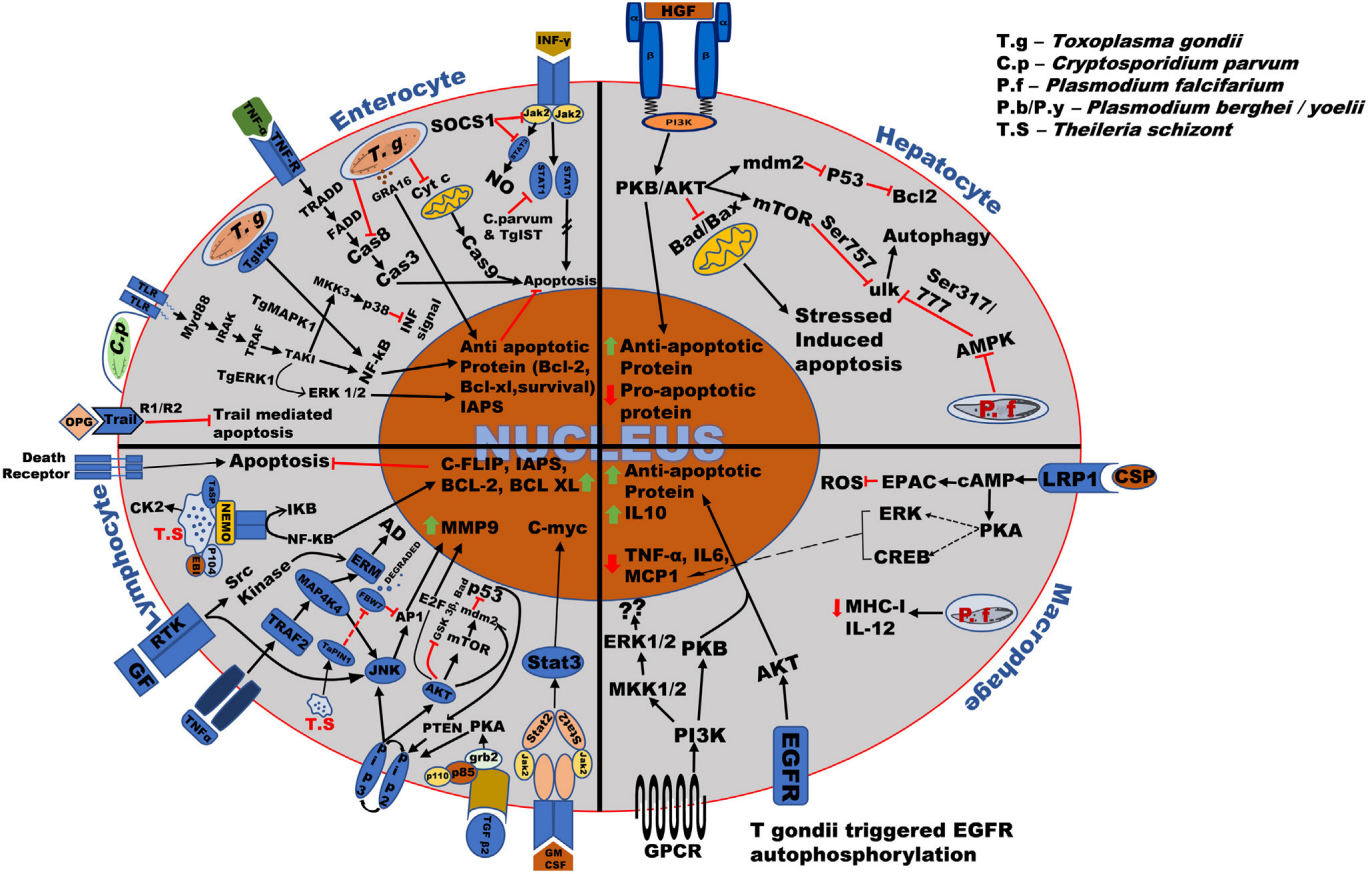
Overall survival mechanism used by the Apicomplexan parasites in different host cells. *Toxoplasma* and *Cryptosporidium* bind to the surface receptor of host cells through the ligands such as EGF, TNF-α, and parasitic surface proteins such as circumsporozoite protein (CSP). After invasion into the host cells such as enterocytes, macrophages, hepatocytes, etc., the parasite modify the host signaling pathway such as TRADD, NF-kB, PKB/AKT resulting in production and upregulation of anti-apoptotic proteins such as Bcl-2, Bcl-xl, and anti-inflammatory cytokines such as IL-10 thereby stopping cytochrome-*c* (Cyt-*c*), TNF-alpha-related-apoptosis-inducing ligand (TRAIL) and BAD, BAX production, and ensuring its survival in the host. *Plasmodium* parasite mainly modifies host PKB/AKT signaling pathway causing upregulation of anti-apoptotic protein and downregulation of pro-apoptotic proteins such as BAD/BAX. *Theileria* schizont proliferates uncontrollably within the host macrophages and lymphocytes. Right after invasion, it upregulates anti-apoptotic proteins such as c-FLIP, IAPs, Bcl-2, Bcl-XL, and proto-oncogenic proteins such as C-myc, antiapoptotic genes such as C-FLIP, Bcl-2, and matrix metallo-protein (MMP9) by majorly targeting host signaling pathways such as NF-κB, JNK/AKT, JAK/STAT, phosphoinositide 3-kinase (PI3-K)/MAPK, and TGF-β2. The regulation of these host signaling pathways causes continuous survival and proliferations of the parasite infected cells which are also common in some cases.

The transcellular migration by sporozoites is mediated by the secretion of perforin protein SPECT or perforin like protein1 (PfPLP1), which is also demonstrated to be important in cell traversal, to perforate the hepatocytes (12, 29). Hepatocytic growth factor (HGF) is released by the perforated hepatocyte (30, 31), which activates c-MET receptor tyrosine kinase (c-MET RTK) on them resulting in the activation of tyrosine residues at the cytoplasmic domain of the c-MET receptor (32, 33). This recruits phosphoinositide 3-kinase (PI3-K) which phosphorylates and sequesters proapoptotic proteins of the BCL-2 family (Bad, Bim, PUMA) through AKT (32–35). AKT, which activates anti-apoptotic proteins (BCl-2, BCL-XL, A1), inhibits Bax on the outer mitochondrial membrane and hinders the permeabilization of the mitochondrial membrane and the subsequent release of proapoptotic signaling molecule such as cytochrome-*c* (Cyt-*c*) and eventually blocks apoptosis (Figure 2) (12, 27, 32, 33).

Once the parasite has already invaded the hepatocyte, host cell apoptosis block is independent of the PI3-K pathway. It seems that the direct intervention of parasite proteins is necessary for modulating the host survival signal (36). One such example is hypoxia mediated by host-dependent HIF-α through AMPK activation which promotes proliferation and parasite survival in the liver (37). The presence of the autophagy marker Atg8 on *P. falciparum* might suggest the parasite’s involving degradative functions, but it instead majorly contributes toward biogenic process (38, 39). After the exoerythrocytic merogony, they trigger apoptosis, although this does not seem to occur by activating the caspase-dependent pathway nor *via* the expression of phosphatidylserine (36, 40). However, a serine-repeated antigen (SERA), a cysteine protease identified in *Plasmodium berghei*, is upregulated and is reported to be playing a role in parasite-induced cell death, parasitophorous vacuole (PV) disruption and merosome formation at the time of exoerythrocytic merozoite egress (12). It suggests that the parasite secretory proteins are mediators of host cell apoptosis in the late liver stage. Among seven calcium-dependent protein kinases (CDPK1–7) known in *Plasmodium*, inhibition of PfCDPK5 leads to schizont stage arrest (20).

Erythrocyte invasion of *Plasmodium* occurs in two stages. First, the interaction of merozoite with the erythrocyte causes host cytoskeletal distortion with the help of high Ca^2+^ level, increasing the contact area between the two favoring merozoite entry and the alignment of its apical pole (14, 19, 20). The role of calcineurin (CnA, CnB) has been implicated in merozoite attachment to erythrocytes, which when knocked down results in impaired invasion (20, 41). Furthermore, DOC2 activation induces microneme secretion of erythrocyte binding antigen (EBA175) and AMA1 (microneme apical antigen1), which are involved in the attachment (20, 42) (Figure 1). At the second stage of invasion, AMA1, in association with RON2 (rhoptry neck protein), binds to the erythrocyte ligand resulting in the formation of tight junction *via* TRAP (43). It has also been observed that the localization of formin at the apical pole nucleates the parasite F-actin with its FH2 domain and helps in parasite motility (44). The forward propulsion of the actin-myosin filament helps in the invasion of merozoite and encapsulation into the PV in the host cytoplasm (14, 45–47). *Plasmodium* invasion entails increased erythrocyte membrane permeability in order to gain nutrients from the extracellular fluid for its survival (45, 48) and further, utilizes the NF-kB-dependent pathway to inhibit host cell apoptosis (49). Following the invasion, the parasite secretes proteins essential for survival, cell adhesion, and pathogenicity. These are transported from the cytosol to the plasma membrane through vesicular transport. The interplay of protein export elements (PEXEL) and *Plasmodium* translocon of exported proteins (PTEX), cause the exported proteins to be transported to the plasma membrane of the RBC (50–52).

Cell cytolysis and infecting new cells are an essential strategy associated with disease progression in *Plasmodium*. Parasite egress is a tightly regulated proteolytic activity of parasite proteases PfSUB1 and dipeptidyl peptidase 3 (DPAP3) in response to increased Ca^2+^ level in the cytosol of the parasite. The PfSUB1, an exoneme secreted protease acts on the PV and the plasma membrane of RBC causing cell rupture and egress (53, 54). Millholland et al. 2013 showed the role of host calpains in cytolysis. Krebs cycle intermediates, formed during replication, act through host GPCR, diffused from host plasma membrane to the PV, also help in cytolysis. The ligand activation of GPCR signal through Gαq activates PKC *via* PLC activation. PKC phosphorylates and liberates adducin (a protein that maintains cytoskeletal integrity) resulting in host cell cytoskeletal deformation, which in turn opens the TRPC6 cation channel on the plasma membrane of RBC. The influx of Ca^2+^ from the extracellular fluid activates CaMK (Ca^2+^/calmodulin dependent kinase) *via* calcium-dependent calmodulin, which phosphorylates host cytoskeletal substrates also resulting in a rapid influx of Ca^2+^. This Ca^2+^ activates and releases host calpain, which causes lysis and dissolution of host cytoskeleton facilitating parasite release (55, 56). The modulation of host survival signaling by *Plasmodium* allows them to successfully establish a specific environment where they can proliferate and differentiate leading to pathogenesis.

Despite substantial progress in the malaria research, restraining the disease still remains a challenge. *P falciparum* parasites owing to their multiple forms/stages, antigenic polymorphisms and AT-rich genome have further impended the problem. Current chemotherapy is based on using artemisinin and artemisinin-based combination therapies (ACTs), however, reports of drug resistance have already emerged. An effective vaccine should be the ultimate goal for long-term control of the disease. To date only the RTS, S/AS01 vaccine, targeting the CSP 178 of *P. falciparum* has reached phase three trials, but has not shown much efficacy (57). There are still many gaps in the understanding of the invasion process of *P. falciparum*, such as which molecules signal the release of Ca^2+^, which leads to adherence and invasion by activating many pathways. These pathways playing role in Ca^2+^ release can be targeted for identifying the novel antigens for developing future vaccines and therapeutics.

## *Babesia*: Malaria Like Parasite

Babesiosis is a hemolytic disease prevalent in tropical and subtropical parts of the world with a broad host range. *B. microti and B. divergens* infect humans and have emerged as a public health concern predominantly in the United States and Europe, respectively (58). Human cases of babesiosis have surfaced recently in Asian countries, including India and Korea (59). *B. bovis and B. bigemina* both infect cattle but, however, higher morbidity and mortality are associated with *B. bovis* resulting in a huge economic loss (60). There are very limited studies investigating the parasite and its host interactions as compared to the other apicomplexan parasites. Owing to the striking similarity between *Babesia* and *Plasmodium* (61), the disease pathogenesis is considered to be similar to malaria during infection in cattle (60).

*Babesia* multiplies in the host erythrocyte in a similar fashion to *Plasmodium*, and they are transovarially transmitted in the vector except for *B. microti* (Table 1) (62). The life cycle of the parasite begins with tick feeding on host blood and simultaneously releasing sporozoites into the host bloodstream (63). Invasion occurs in a similar fashion as observed for *Plasmodium, Toxoplasma*, and *Cryptosporidium via* gliding mechanism using microneme and rhoptry secretions (61, 64). Parasite loosely attaches to the surface of the host RBC by its surface GPI anchored proteins and within the apical secretory organelles. Variant erythrocyte surface antigen 1 (VESA1), a heterodimeric protein of *Babesia* is known to play role in cytoadherence to the host erythrocyte surface (62, 65). After entry of the sporozoites into the red blood cells, they divide by binary fission and produce merozoites. Erythrocyte lysis further allows each merozoite to invade a new RBC and successive merogonies follow (62, 64, 66, 67). Merozoites interact with the RBC surface receptors and play a major role in invasion.

In *Babesia*, the role of Ca^2+^ has been primarily described in invasion and egress mechanism of the parasite, however, the modulation of the host signaling pathways are not thoroughly understood. In *B. bovis*, Ca^2+^-dependent protein kinase inhibitor showed growth limiting effects (68), though, in *B. divergens*, it impacted egress of the merozoites from erythrocytes (69, 70). Since there is no PV formation in *Babesia*, less Ca^2+^ is released during egress as compared to *P. falciparum* parasites (Table 2) (70, 71).

**Table 2:** A number of host signaling pathways modulated by *Theileria, Plasmodium, Babesia, Toxoplasma, and Cryptosporidium* during its invasion, survival, expansion, and egress in the host cell.

Host factor	Parasite	Mechanism	Benefit to parasite	Reference
**Cytoskeletal remodeling**				
Actin	Theileria Plasmodium Toxoplasma Cryptosporidium	Actin rearrangement through ERM proteins Parasite formin-mediated F-actin nucleation *Via* F-actin and Arp2/3 recruitment. Activating Arp2/3 *via* c-src kinase and phosphoinositide 3-Kinase (PI3-K).	Helps in cell motility and dissemination Key event for Parasite motility/invasion of erythrocytes. Parasite motility and entry. Parasite entry Helps invasion	Baumgartner et al. (72) Baum et al. (44) Bargieri et al. (43) Gonzalez et al. (73) Chen et al. (74)
**Ca^2+^ signaling**				
Ca^2+^	Theileria Plasmodium Toxoplasma Cryptosporidium Babesia	Intrasporozoite calcium Mobilization of intracellular Ca^2+^ Mobilization of extra/intracellular Ca^2+^ PKCα depended on leaky tight junctions Mechanism unknown	Favors internalization Helps invasion Microneme secretion required for cell motility Favors invasion Parasite entry and egress	Shaw (75) Gao et al. (42) Lourido and Moreno (21) Hashim et al. (76) Mossaad et al. (70)
**Survival or apoptosis**				
NF-kβ	Theileria Plasmodium Toxoplasma Cryptosporidium	Direct activation through IKK recruitment Activated by infected erythrocyte Activated either by host or parasite IKK Parasite-induced activation	Helps survival Helps survival by upregulating anti-apoptotic pathway Helps survival Helps survival	Heussler et al. (77) Tripathi et al. (49) Molestina and Sinai (78) Chen et al. (79)
PI3-K	Theileria Plasmodium Toxoplasma Cryptosporidium	Activated *via* TGF-β2 receptor Activated *via* c-MET receptor tyrosine kinase receptor in hepatocytes Activated *via* Gα_i_-PCR (Protein Coupled Receptor) Recruitment of PI3-K by sporozoite attachment	Promotes survival *via* inhibiting host apoptosis Helps survival Promotes survival *via* inhibiting host apoptosis Helps in invasion	Haidar et al. (80) Rodrigues et al. (33) Kim (81) Chen et al. (74)
JAK/STAT	Theileria Toxoplasma Cryptosporidium	Activated *via* Granulocyte-macrophage colony-stimulating factor (GM-CSF) autocrine signaling Prolong phosphorylated state of STAT3/6. STAT1 inactivation by T. *gondii* inhibitor of STAT1 (TgIST) Inhibited *via* STAT1 α depletion	Promotes proliferation *via* enhancing host c-myc levels Promotes survival *via* limiting IL12 and IFNγ. Promotes survival *via* preventing IFNγ signaling. Promotes survival *via* inhibition of NO production	Dessauge et al. (82) Laliberté and Carruthers (83) Olias et al. (84) Lean et al. (85)
p53	Theileria Plasmodium Toxoplasma	Sequestration of p53 and degradation Mdm2-mediated p53 inhibition GRA16-mediated p53 regulation	Aids survival Promotes liver stage infection Benefits the parasite by altering p53 levels.	Haller et al. (86) Kaushansky et al. (87) Bougdour et al. (88)
**MAP kinase pathway**				
JNK	Theileria Toxoplasma Cryptosporidium	Activated *via* grb2 association with TGF-β2 JNK is inhibited JNK is inhibited	Promotes survival and metastasis. Escaping JNK-mediated apoptosis Escaping JNK-mediated apoptosis	Lizundia (89) Kim (81) Liu et al. (90)
p38 MAPK	Toxoplasma Cryptosporidium	IFN-γ signaling-mediated production of iNOS is inhibited. Induces NETosis	Facilitates survival Killing of parasite	Brumlik et al. (91) Muñoz-Caro et al. (92)
ERK1/2	Toxoplasma Cryptosporidium	Activated *via* TgERK7 Parasite-induced NETosis	Ensures survival and reinfection Favors killing of parasite	Li et al. (93) Muñoz-Caro et al. (92)
**Autophagy**				
	Theileria Plasmodium Toxoplasma	Inhibits AKT-activated mammalian target of rapamycin inhibits autophagy *Via* EGFR/AKT pathway	Promotes survival Promotes liver stage infection Helps parasite bypass autophagy	Duszenko et al. (94) Kaushansky et al. (87) Muniz-Feliciano et al. (95)
**Cellular metabolic stress**				
Reactive oxygen species (ROS)	Theileria Plasmodium Toxoplasma Cryptosporidium Babesia	Activates NF-kβ and PI3-K signalling pathways ROS accumulation in RBCs Alters ROS levels by downregulating nox4 and inhibiting p38. Scavenges ROS by parasite peroxidase Scavenges ROS by parasite peroxidase	Promoting survival Inhibit parasite growth Promotes survival Promotes survival Promotes survival	Metheni et al. (96) Usynin et al. (17) Zheng et al. (15) Treeck et al. (97) Hong et al. (98) Bosch et al. (99)
NOS	Theileria Plasmodium Toxoplasma Cryptosporidium Babesia	Upregulation of iNOS by NF-kβ Infection-mediated upregulation of iNOS TgMAPK1-mediated NO reduction Inhibition of IFN-γ-mediated NO upregulation IFNγ-mediated upregulation	Promotes survival *via* NO-mediated inhibition of Fas apoptosis Parasite clearance Promotes survival Promote survival Parasite growth arrest	Durrani et al. (100) Chiwakata et al. (101) Brumlik et al. (91) Lean et al. (85) Goff et al. (102)
Hypoxia	Theileria Plasmodium Toxoplasma	Induces transcription of proteins required for the metabolic shift HIF-induced AMPK activation Protects HIF1α degradation and enhanced HK2 expression	Enhances survival Promotes development of exoerythrocytic forms (EEF) and increases iron uptake Promotes parasite growth *via* increasing glycolytic flux	Metheni et al. (103) Ng et al. (37) Menendez et al. (104)
**Cytolysis and egress**				
GPCR	Toxoplasma and Plasmodium	PKC-mediated Ca^2+^ influx, finally activating calpain which proteolyse host cytoskeleton.	Parasite egress	Chandramohanadas et al. (105) Millholland et al. (56)

Transovarial transmissions in tick vector and straight entry of sporozoites into erythrocytes are some key features, which make *Babesia* parasites distinct from *Plasmodium* or *Theileria* parasites. Few studies to understand the mechanism of disease pathogenesis during *Babesia* infections have been published. Also, the mechanism of entry and transmission of the parasites are poorly defined. It will be important to investigate the parasite invasion and evasion strategies along with parasite vector interactions for identifying key genes that might play an important role in immune evasion or disease pathogenesis.

## *Theileria*: A Livestock Pathogen

*Theileria annulata and T. parva* cause tropical theileriosis and east coast fever, respectively, in ruminants predominantly in cattle causing enormous economic loss to the livestock industry (106, 107). Tick vector transmits the parasite upon feeding on animal through the saliva (108–110). After entering the blood stream, it infects WBC of different lineage, *T. parva* infects B cells and T cells whereas *T. annulata* infects B cells and cells of monocyte lineage. The sporozoites, i.e., the infective stage of the parasite passively invade the host cell by zippering mechanism, which is unlike other apicomplexan discussed in which a tight continuous junction is formed between the host cell surface and the parasite sporozoites (108). In addition, the role of MHC class I molecule (Figure 1) (111), intrasporozoite Ca^2+^ and protein kinases of host and parasite and the G-protein linked signaling has been shown in invasion (75, 112). After entry into the host cell, parasite rhoptries and its microsphere discharges dissolve the enveloping PV membrane (108), and move to the host cell cytoplasm rather than to PV in comparison to other apicomplexan parasites and provides it with an advantage of escaping lysosomal degradation (Table 1). Additional advantage of staying in the host cytoplasm allows the parasite to modulate several signaling pathways, such as TGF-β, JNK, PI3-K, NF-κB, src kinase, and casein kinase 2 (CK2) (80, 100, 113–115).

*Theileria* transforms their host cell into a cell with a cancerous phenotype by modulating several host cell kinases and activating transcription factors (116). Several studies have been done to identify parasite protein instigating epigenetic changes that may lead to successful transformation. *T. annulata* protein, TaPIN (secretory prolyl isomerase Pin) has been reported to promote transformation by degrading FBW7, a host ubiquitin ligase *via* stabilizing c-JUN (117). p104 and TaSP are surface proteins which have been reported to be phosphorylated in a host cell cycle-dependent manner and might be involved in transformation (118). Two more proteins are TashAT group of protein, which contains AT hook DNA-binding motif and nuclear localization signal and found to be localized in host nucleus (119). *SuAT1*, a parasite gene, contains AT hook DNA-binding polypeptide and predicted signal peptide, PEST motifs and nuclear localization signals, which may interact with the host cell and play a role in transformation (120). Studies to identify epigenetics changes are scarce, only one study has shown the role of oncomiR mir155 in repressing DET1 protein (involve in c-Jun ubiquitination) and stabilizing c-Jun (121). The parasite schizont hijacks the host mitotic assembly resulting in its clonal expansion (122–124).

*Theileria* transformed cells can be reversed, unlike tumor cells upon treatment with BW720c (122). The transformation occurs by modulating several signaling pathways which ultimately inhibits apoptosis, increases proliferation, and encourages metastatis (116). NF-κB is constitutively expressed in *Theileria* infected cells, which in turn upregulates many anti-apoptotic proteins, such as c-FLIP, IAPs, Bcl-2, and Bcl-XL, and induces Gadd45β that blocks the pro-JNKK2-mediated apoptotic JNK pathway. NF-κB is activated by recruitment and phosphorylation of IKK signalosome α and β subunits, which further phosphorylates inhibitory κB (iκB) setting NF-κB free to translocate to the nucleus (125). Infected cells release a plethora of cytokines and growth factors that activate TGF-β receptor (I and II) and TNF-α receptor (126). TGF-β2 activates smad2/3 and subsequently smad4, which over-expresses COX-2 resulting in increased levels of prostaglandins and downregulates PKIG, a potent inhibitor of PKA pathway. Simultaneously, TGF-β2 is accounted for parasite motility and invasiveness by activating Rho–ROCK kinase and recruiting an adaptor protein growth factor receptor-bound protein 2 (Grb2) to TGF-RII receptor. The signaling descends by Grb2, activating downstream PI3-K/AKT and JNK pathway (80, 127). Activator protein 1 (AP1), a JNK activated transcription factor drives B cell integration cluster (BIC) transcription upregulating miRNA 155 which inhibits DET1 resulting in accumulation of c-Jun and increased proliferation (89, 121, 128–131). B-1 a bovine analog of MMP9 (ECM degrading proteinase) containing AP1 binding sites also helps in detachment and metastasis of the infected cell to other organs (89, 132, 133).

*Theileria* modulates the host PI3-K/AKT pathway to be regulated by granulocyte-macrophage colony-stimulating factor (GM-CSF), depending on an autocrine loop and, hence, sharing a major role in cell proliferation (134, 135). Phosphorylation of the AKT protein by class I PI3-K facilitates the release of Rb bound E2F transcription factor, activating MDM2 (E3 protein ubiquitin ligase and negative regulator of p53 tumor suppressor gene) directly or through mammalian target of rapamycin (mTOR). AKT-mediated inhibition of several proapoptotic genes (*bad, foxo*) and GSK-3β help the infected cells to combat the stress-induced mitochondrial-mediated apoptosis and to maintain elevated c-Myc levels, respectively (Table 2; Figure 2) (136). Hypoxia-inducing factor (HIF-1α) is activated by mTOR pathway and by constitutive NF-κB and AP1 production (137). Increased levels of ROS during infection and HIF-1 expression induce the Warburg effect allowing the parasite to establish uncontrolled proliferation (96, 103, 138, 139). PI3-K/AKT pathway, therefore, plays a very important role in survival and proliferation as well as in metastasis of *Theileria*. PTEN, an inhibitor of the PI3-K pathway and activator of p53 are suppressed majorly by NF-κB and CK2 (82, 140, 141). CK2 also dampens TNF/Fas-mediated apoptosis and accelerates iκB degradation augmenting NF-κB activation. Activation of JAK/STAT pathway *via* GM-CSF autocrine loop enhances c-myc expression, whereas phosphorylation by CK2 stabilizes this potent oncogene primarily upregulated in *Theileria* infected cells (Figure 2) (82, 115).

The proliferation of infected cells is followed by evasion and metastasis, which involves cytoskeleton alteration. TNF-α binding to its receptor recruits TNF receptor-associated factor 2 (TRAF2), which may either activates NF-κB or a mitogen-activated protein kinase, MAP4K4 (134). MAP4K4 bifurcates into JNK activation and ERM (ezrin/radixin/moesin) cytoskeletal protein phosphorylation, helping in actin dynamics through Rho kinase. ERM may also be activated through src kinases (72). Interfering with autophagic mechanisms also augments survival of the parasite within the cell. Dampening peptide presentation by CD4^+^ and CD8^+^ infected cells is a way opted by the parasite to increase its chances of survival enhancing the establishment of infection (94). In contrast to other apicomplexan parasites, *Theileria* usually does not egress and remains attached to the host mitotic assembly. Although under unfavorable circumstances the schizont ruptures releasing merozoites, which invade RBCs, forming piroplasm, which is taken up by the tick and the cycle resumes. *Theileria* parasites have thereby evolved a wide range of strategies to help them survive and proliferate inside the host cells.

*Theileria* parasites are considered as the smartest among the apicomplexan group for their ability to manipulate the host cells. However, the parasite proteins and molecular mechanisms behind the host cell manipulation are still not clear. Very few proteins are identified till now, which are involved in the host–parasite interaction. The presence of a heterogeneous population of *T. annulata* parasite strains in the field is making it difficult for a currently used attenuated vaccine (schizont stage) to be effective against *T. annulata* parasites. Resistance against burpaquone, a drug currently being used for the treatment of theileriosis, and acaricide, used for controlling the tick vector, has hampered the control of the disease. In this post genomic era with the availability of advanced genomic and proteomic tools, better studies are needed to dissect the pathways modulated by *Theileria* in detail and select new targets for the development of second-generation drugs and vaccines for disease control.

## Enteric and Zoonotic Apicomplexan Pathogen

### *Toxoplasma* 

Toxoplasmosis, caused by *Toxoplasma gondii*, is one of the most well-studied zoonoses (142). *Toxoplasma* appears to be one of the most feared apicomplexan parasites due to a considerable number of congenital transmission incidents and subsequent fetal damage in animals and humans (143, 144). It also causes neurologic deficits (145) and chorioretinitis (146). The life cycle of the parasite circulates between definitive (feline) and intermediate hosts (mammals/birds, etc.). Sexual phase occurs in the small intestine of the feline host from which the oocysts/tissue cysts are excreted along with the feces and ingested by the intermediate host through multiple routes. The oocysts release sporozoites, which invade the intestinal lining. On the other hand, tissue cysts release bradyzoites which differentiate into tachyzoites (147–149). The tachyzoites further replicate in the host and may again differentiate into bradyzoites in the brain, liver, and muscle tissue forming cysts (147, 150, 151). This inter-conversion between tachyzoites and bradyzoites appears essential to the life cycle and infective potential of the parasite (Table 1) (152).

Host–parasite interactions are mostly *via* secreted parasite proteins from their rhoptries, micronemes, and dense granules, which help parasite in cell invasion, survival, and egress. Invasion by *T. gondii* involves gliding mobility prompted by an actin-myosin motor based complex (153) and interrelated signaling cascades as well. The parasite attaches loosely with the host cell surface *via* GPI-linked proteins, surface antigens (SAGs), SAG-related sequences (SRSs), and SAG unrelated surface antigens (SUSAs) (154, 155). After the secretion of *Toxoplasma* microneme adhesion proteins (MICs) into the host, there is an increased activity of Calcium/Calmodulin (Ca/CAM)-dependent processes leading to the secretion of Phospholipases (sPLA2 and PLA2) (Figure 1). *T. gondii* calcium-dependent protein kinase1 (TgCDPK1) has been reported to be involved in microneme secretion and can thereby regulate cell motility which is essential for invasion (156). sPLA2 secretion causes the release of microneme proteins MIC3/MIC2, which induces Ca^2+^ release from host ER *via* Protein Kinase C-Inositol 1,4,5 triphosphate (PKC–IP3) pathway. cPLA2 activated by parasite MAPK causes membrane fluidification by hydrolyzing host membrane phospholipids (157). Therefore, Ca^2+^signaling induce a lot of complex cascades facilitating parasite invasion (Figure 1; Table 2). Apicomplexan have been reported to have several Ca^2+^ATPases and CA^2+^/H^+^ exchangers, which help in invasion (21).

*Toxoplasma* invades *via* gliding movement, which results in actin remodeling by F-actin ring formation at the point of entry with the subsequent recruitment of Arp2/3 complex (Figure 1) (73, 158). Parasite rhoptries are secreted followed by microneme secretion, which consists of RON and traditional rhoptries proteins (ROP). RON2 and AMA1 associate together to form a tight junction between the host and the parasite referred to as moving junction (MJ) facilitating the formation of PV (159–161). Sporozoites also invade using paralogs of AMA1 and RON2 conveniently named as sporoAMA1 and sporoRON2 (162). Traditional rhoptry proteins such as Rop 17, Rop 18 (kinase), and Rop 5 (pseudokinase) reside on the PV membrane inhibiting the accumulation of immunity-related GTPases (IRGs). This complex also has a dense granule protein, namely GRA 7 which has a definite impact on IRG turnover (163, 164).

Once inside the host cell, the parasite thrives on host nutrients by expressing various parasite transporters, enzymes, and following complex cascades (165). *T. gondii* inhibits apoptosis and dodges autophagy by manipulating PI3-K pathway, the immediate downstream effector protein kinase B (PKB/Akt), JAK/STATs, mTOR, NF-kβ, ERK1/2, C-myc, and microRNAs to promote its survival (165, 166). The parasite avoids lysosomal degradation by cleverly maintaining the non-fusogenic nature of the PV. Studies suggest that *T. gondii* micronemal proteins (MICs) with epidermal growth factor (EGF) domains activate Epidermal Growth Factor Receptor (EGFR) on endothelial, retinal cells, and microglia keeping the parasite protected in the vacuole (95). It has been reported that the parasite causes mTOR activation in an infected host cell even in the absence of phosphorylation of 4E-BP1 and S6K1 (167). Later, a study confirmed the role of mTORC1 and C2 in host cell invasion and persistence of infection (168).

*Toxoplasma gondii* disrupts host apoptotic pathways primarily by affecting the release of Cyt-*c* and thereby preventing activation of caspase 3 (166). The parasite modulates the host NF-kβ pathway in line with *Theileria* causing increased expression of anti-apoptotic genes, although, there is also a role of *Toxoplasma* IKK (TgIKK) in maintaining the NF-kβ response which declines after the initial activation by the host IKK (78). A dense granule protein GRA 15 also activates this pathway *via* TNF receptor-associated factor 6 (TRAF6) thereby inducing the release of pro-inflammatory cytokines (169). Rop 16, on the other hand, might be playing a role in inhibiting cytokine synthesis by host macrophages (170). Hence, opposed the effect of two of these factors determines macrophage polarization in the host (171). *Toxoplasma* polymorphic effectors determine macrophage polarization and intestinal inflammation (171) *Toxoplasma* inhibits the proapoptotic genes (BCL-2, Bad, caspase-9) by modulating the host–PI3-K pathway. It also hinders apoptosis by downregulating phosphorylated c-Jun N-terminal kinase levels (81). AKT/PKB pathway is upregulated, which serves the parasite by inhibiting forkhead transcription factor (FKHRI) resulting in decreased levels of proapoptotic factors, such as Bim and FasL (172). Infected cells also display increased expression of anti-apoptotic proteins, such as BCL-2, BFL1, BCL-XI, BCL-W, and MCI-I, and reduced expression of proapoptotic factors Bad and Bax (Figure 2).

*Toxoplasma* ERK-7 (TgERK7) protein has been recently demonstrated to play an important role in the intracellular proliferation of the parasite in the host (93). *T. gondii* also protects itself from host interferon-γ (IFN-γ)-mediated pathway by obstructing the expression of IFN-γ activated genes. *T. gondii* inhibitor of STAT1 (TgIST) has been shown to bind to activated STAT1 in the host cell membrane and recruits host Mi2/NURD complex which keeps the STAT1 in inactivated stage thereby preventing pro-inflammatory gene expression (84, 173). Elevated Ca^2+^ during T. *gondii* infection activates the protein kinase C cascade which further activates COX-2 resulting in increased prostaglandin E2 (PGE2) levels helping in the resolution of inflammation (174). Furthermore, a dual role of TGF-β during infection by inducing or suppressing the immune system has been reported (175).

During infection, TRAF6 is activated by the parasite dense granule protein GRA7 which leads to unusually increased levels of ROS in the cells (97). Increased levels of ROS, few cytokines and growth factors, causes elevated HIF-1 levels *via* dampening prolyl hydroxylase domain containing protein 2 (PHD-2) levels downstream to Type I TGFβ receptor signaling. Influencing the levels of such a crucial host factor as PHD-2 is pivotal for the maintenance of a secure haunt of the parasite (176). A host kinase-HK2 also activates HIF-1 expression resulting in glycolytic flux and Warburg effect, as identified by siRNA screening (104). A microarray-based study reveals that increased HIF-1 level in infected cells lead to activation of EGR1 and AP1 which play roles in inducing resistance against drugs and proliferation, respectively (177, 178). The parasite also seizes the IFN-γ-induced iNOS production by *T. gondii* expressed MAP kinase (TgMAPK1), which reduces NO production by p38 MAPK (91). But on the other hand, a dense granule protein GRA24 also plays a role in maintaining p38α autophosphorylation, forming a complex, which consequently activates EGR1 and cFOS which induce the release of MCP-1 and IL-12, which can keep the parasite load in check (179).

*Toxoplasma* also modulates p53 levels for its own benefit by GRA16, another parasite dense granule protein which binds to two host enzymes-HAUSP and PP2A phosphatase in the host nucleus (88). *T. gondii* reportedly alters dopaminergic and GABA-ergic signaling due to elevated levels of mi-RNA132 which might be the underlying cause for the neuronal abnormalities often found associated with the infection (180). The parasite utilizes GABA to partially satisfy its carbon requirements and also in egress (181). Once it has successfully established infection, egress mainly occurs *via* GPCR-coupled signaling pathway similar to *Plasmodium*. TgCDPK1 and TgCDPK3 activated by Ca^2+^ influx have been reported to play a role in egress. Studies suggest that a parasite pore forming protein TgPLP1 might be responsible for making the PV perforated to make egress easier (182). Recently, cGMP-dependent PKG has been identified to play an important role in controlling egression (156).

As it appears from the above discussion, the cunning parasite can steer a staggering number of host signaling pathways in direction of its own purpose. However, despite the fact that very specific knowledge is available about particular such proteins, it is not clear how they affect host gene expression since such nucleus targeted proteins do not really resemble host transcription factors neither can they bind to the host cell DNA (164). Interestingly, not all of these secreted proteins benefit the parasite. Some actually trigger the host immune system to call up its guards. Now, how might the parasite strike a balance to sustain infection or how might we use such kind of knowledge to limit infection still remains to be worked out.

### *Cryptosporidium* 

*Cryptosporidium* commonly causes gastrointestinal diseases worldwide, which albeit minimally invasive in the immunocompetent host (both human and animals) can be deadly in immunosuppressed patients (183, 184). *C. parvum* with a broad host range and zoonoses is considered a more important pathogen in comparison to *C. hominis*, which only infects human. The disease prevalence ranges from 1 to 37% in countries such as Africa, Asia, Australia, South America, and Central America (185, 186).

Its life cycle comprises of a sexual and an asexual stage, which takes place in a single host (187). Similar to *Toxoplasma, Cryptosporidium* infection occurs by ingestion of oocysts through contaminated water followed by excystation and release of sporozoites. These zoites then invades the enterocytes by gliding movement (Table 1, Figure 1) (188). *Cryptosporidium* form an intracellular but extra cytoplasmic PV wherein they get developed into spherical trophozoites (184, 189). Invasion of host epithelial cells occurs *via* aggregating the host actin and actin binding protein, villin at the site of parasite attachment and further inducing host tyrosine kinase signaling cascades (189, 190). Reports of numerous *C. parvum* proteins have been implicated in attachment, invasion, and intracellular development (191, 192). p30, a galactose-*N*-acetylgalactosamine (Gal/GalNAc) lectin parasite protein has been identified which forms an adhesion complex along with gp40 and gp900 (193). Furthermore, the cryptosporidial binding leads to the formation of sphingolipid-enriched membrane microdomains which attracts Gal/GalNAc epitope containing glycoproteins on the host membrane parasite interface, activating PI3-K (192). The PI3-K cascade successively activates Cdc42, N-WASP, and Arp2/3 (actin-related protein 2/3) resulting in the formation of actin plaque (74, 124, 194). The parasite recruited src tyrosine kinase subsequently phosphorylates cortactin stimulating the polymerization and rearrangement of the actin cortex in the cell periphery through activation of Arp2/3 complex proteins (Figure 1) (74, 195). Increase in local cell volume by accumulation of host aquaporin AQP1 and Na+/Glucose co-transporter also aid in efficient membrane protrusions (196). Few studies have also shown the role of host calpain in remodeling host cytoskeleton which is essential during parasite invasion (197).

Ca^2^*^+^*-ATPase located at the *Cryptosporidium* sporozoites apical and perinuclear regions helps it in fulfilling its Ca^2^*^+^* requirement during the invasion (193). *Cryptosporidium* also possesses 7 CDPKs, which has a role in invasive and regulatory processes similar to *Plasmodium* and *Toxoplasma*. *Cryptosporidium* invasion is promoted by a Ca^2^*^+^*-dependent PKC signaling pathway, which disrupts the cell–cell junction. PKC causes downstream activation of PKCα which has been associated with tight junctional leakiness in renal epithelial cells (76, 193, 198). *Cryptosporidium* embodies a novel Ca^2^*^+^*-activated nucleoside diphosphatase (apyrase, CApy), which interfere with extracellular nucleotide and modulates inflammatory pathways delaying the response against parasite clearance (199). The trophozoite stage of the parasite inhibits apoptosis; however, schizont- and merozoite-affected cells are handled by host apoptosis through Fas/FasL signaling (200, 201).

*Cryptosporidium* activates NF-κB pathway by inducing IL-8 secretion and acting synergistically with AP1 and IL-6 (79) (Figure 2; Table 2). It has also been reported to play a role in activating other survival signals, e.g., over expression of antiapoptotic proteins (bcl-2, IAP, survivin) and inhibition of proapoptotic proteins (bax) (77, 202). Myc, an oncogenic protein plays a role in positive regulation of parasite survival, whereas PTEN, an inhibitor of PI3-K, negatively regulates the anti-apoptotic protein (90). Also, microarray analysis revealed that TNF-superfamily receptor osteoprotegerin (OPG) is upregulated in infected host intestinal mucosa by microarray. The overexpression of OPG helps in evading host defense by inhibiting TNF-alpha-related-apoptosis-inducing ligand (TRAIL)-mediated apoptosis and supporting the parasite to complete its life cycle (184, 203). The host tries to control the propagation of *Cryptosporidium* by enhancing Th1 response characterized by the production of IFNγ and IL-12. The parasite, too, in turn, erodes the JAK/STAT-mediated IFNγ signaling by depletion of STAT1-α (85, 204). TNF-α and TGF-β play roles in providing the host protective immunity and healing effect against the infection (85, 205, 206). Again, increased mucin levels in the host by COX-2-mediated PGE2 protects the host (183), ERK1/2 and p38 MAPK pathway also assist the host cells to destroy the parasite by inducing NETosis (Formation of the neutrophil extracellular trap) (92).

Absence of Apicoplast in *C. parvum* parasites and complications in their *in vitro* propagation has posed problems for researchers involved in drug or vaccine development. Despite many efforts by *Cryptosporidium* to modulate the host signaling pathways, the parasite loses the battle against the host. At present, only one drug (nitazoxanide) with limited efficacy is approved for treatment of Cryptosporidiosis. Further studies are needed to better understand the egress mechanism of *Cryptosporidium* (74). Susceptibility to the parasite has shown to be linked with the immune status of the host. Understanding the host–pathogen interaction will be critical in designing new tools for effective control of the disease.

## Final Conclusion

A substantial amount of research has been done to gain insights into pathways by which these parasites modulate and undermine the host defense, yet gaps in knowledge still prevail and many questions remain unanswered. In this review, we have attempted to include all the major work carried out in this field. Advancement in gene editing technologies and whole genome sequencing of these pathogens lead us to better understand the manipulation strategies used by the parasites. Emerging problems of either drug resistance or unavailability of an effective vaccine against some of the parasites make the precise comprehension of the sabotage techniques employed by the parasites a primary requisite in order to curb the morbidity rate.

As discussed above, it is apparent that few of the host defense pathways targeted by these parasites to survive and proliferate in the host cell are common among the mentioned pathogens. Host cell invasion by *Plasmodium, Babesia, Toxoplasma*, and *Cryptosporidium* occur *via* gliding movement, whereas in *Theileria* an overall different process known as zippering takes place. *Plasmodium, Toxoplasma*, and *Cryptosporidium* reside and replicate inside the PV in the host, however, *Theileria* and *Babesia* survives in the host cytoplasm. *Theileria* clearly takes an advantage of staying in the host cytoplasm by modulating numerous pathways, though any such information about *Babesia* has to be still investigated. Several pathways are modulated by majority of these parasites such as host cytoskeleton remodeling, Ca^2++^ modulated signaling pathways, and apoptotic pathways which helps in their survival. In the review, we have discussed the cross talks happening between the parasite and the host and observed that the multifaceted nature of the parasite gives them an upper hand over the host.

Future studies focusing on

(i) Exploring the parasite proteins and their role in host–parasite interface interaction will provide in-depth understanding of the invasion process. These targets can be further utilized to develop vaccine or drugs.
(ii) What are the alteration in the host cell that leads to the nutrient acquisition after invasion and the host factors contributing to parasite replication?
(iii) Studies are also required to develop inhibitors against known molecules/pathways, which help the in intracellular survival of parasite in the host cell.

Therefore, targeting the common pathways playing crucial role in all parasites survival and dissemination may be a good approach to understand disease pathogenesis and controlling the disease.

In order to deal with these cunning pathogens, we need all the necessary information to be able to target important molecules for a vaccine or drug development. However, a considerable amount of research and thorough screening of presently available literature is still required to better understand how these parasites exploit their hosts for their own survival. Apicomplexan parasites infecting human beings such as *Plasmodium* is hugely funded and globally studied; however, so is not the case when it comes to parasites such as *Babesia* and *Theileria*, which are of veterinary importance. Therefore, for more in-depth understanding of these pathogens, tenacious research is expected which would only be possible through the combined efforts of researchers and support from funding agencies on a global range.

## Author Contributions

All authors mentioned have made a significant effort and contributed intellectually to the work and approved it for publication.

## Conflict of Interest Statement

The authors declare that the research was conducted in the absence of any commercial or financial relationships that could be construed as a potential conflict of interest.
